# CAM Model: Intriguing Natural Bioreactor for Sustainable Research and Reliable/Versatile Testing

**DOI:** 10.3390/biology12091219

**Published:** 2023-09-08

**Authors:** Carla Palumbo, Federica Sisi, Marta Checchi

**Affiliations:** Department of Biomedical, Metabolic and Neural Sciences, Section of Human Morphology, University of Modena and Reggio Emilia—Largo del Pozzo, 41124 Modena, Italy

**Keywords:** chorioallantoic membrane (CAM), angiogenesis, organotypic culture, engineered 3D scaffold

## Abstract

**Simple Summary:**

The chicken embryo chorioallantoic membrane (CAM) is an in ovo model that has been known for years. It has mostly been used to test the characteristics of molecules and cell pellets and their potential interactions with vessels, particularly in cancer studies. Recently, we repurposed such a model by highlighting its ethical features, because, to a large extent, it can reduce the use of animal experimentation and produce rapid results. Its applications have multiplied in recent years, allowing for the development of more in-depth and comprehensive analyses and, thus, reducing the gap between in vitro and in vivo experimentation. Since the CAM model allows for the replacement, reduction, and refinement of preclinical experimentation (rules of the “3Rs”), it makes experimental research more sustainable and in line with animal welfare. The objective of this review is to illustrate the potential of the CAM assay, with a particular focus on the setup of organotypic cultures. This type of assay may be used as a preclinical model to assay recovery strategies for critically-sized bone injuries, i.e., severe fractures that do not spontaneously heal due to disruption of the vascular network and a large gap between the two bone stumps.

**Abstract:**

We are witnessing the revival of the CAM model, which has already used been in the past by several researchers studying angiogenesis and anti-cancer drugs and now offers a refined model to fill, in the translational meaning, the gap between in vitro and in vivo studies. It can be used for a wide range of purposes, from testing cytotoxicity, pharmacokinetics, tumorigenesis, and invasion to the action mechanisms of molecules and validation of new materials from tissue engineering research. The CAM model is easy to use, with a fast outcome, and makes experimental research more sustainable since it allows us to replace, reduce, and refine pre-clinical experimentation (“3Rs” rules). This review aims to highlight some unique potential that the CAM-assay presents; in particular, the authors intend to use the CAM model in the future to verify, in a microenvironment comparable to in vivo conditions, albeit simplified, the angiogenic ability of functionalized 3D constructs to be used in regenerative medicine strategies in the recovery of skeletal injuries of critical size (CSD) that do not repair spontaneously. For this purpose, organotypic cultures will be planned on several CAMs set up in temporal sequences, and a sort of organ model for assessing CSD will be utilized in the CAM bioreactor rather than in vivo.

## 1. Introduction

The field of clinical research has shown significant improvement in the therapeutic strategies, which are continuously developing to cope with the problems that an aging population imposes. The efficacy and the safety of these strategies need to be evaluated through extensive preclinical testing, including animal experimentation, which is essential for approval from regulatory office such as Food and Drug Administration (FDA) prior to being applied in clinical approaches. These necessities come in contrast with sustainable research; this is why there is a need for the development of alternative strategies to animal testing [1,2].

In recent years, thanks, in part, to some funding agencies that are more attentive to animal welfare, there has been much emphasis on projects that include the development of alternatives to animal experimentation, which, to date, appears to be a mandatory and necessary step for bringing new devices/therapies to market, but which, on the other hand, is too expensive and no longer sustainable. Alternatives proposed in the world of basic and clinical research include, for example, tissue cultures [3,4,5], 3D cultures [6,7,8,9], organoids [10,11], microfluidics such as organs-on-a-chip [12,13,14], and the use of chicken chorioallantoic membrane [15,16,17,18,19,20,21]. The latter is a technique that was developed in the past for angiogenesis assays because of its highly vascularized system, ease of use, inexpensiveness, and great ethical value. In the last decade, chorioallantoic membrane (CAM) assays have been re-discovered and used not only for traditional angiogenic assays [22,23] but also as non-innerved bioreactors and providers of rapidly growing vascular beds that mimic the blood supply for organ culture [24,25,26,27]. In addition, the embryo is not immunocompetent until 16–17th days of development; therefore, it cannot sustain rejection reactions [28,29,30]. The versatility of this model has made it widely used in recent years; in fact, this has opened new pathways to more sustainable, ethical, and animal-welfare-supportive research. 

In particular, the field of bone regenerative medicine can benefit, for example, from the use of organ culture models with CAM. A fundamental prerequisite for the formation of new bone is the presence of a well-developed vascular bed that serves as a template for the generation of new bone thanks to the collaboration of the bone-forming cells [31,32,33,34,35,36]. The study of proangiogenic potential and its associated implications for tissue regeneration require complex in vivo models comprising all steps of the angiogenic process. The CAM model offers a simple, easily accessible, and inexpensive angiogenic screening tool compared to other animal models. In addition, the great ethical value of this in ovo model lies in the application of the 3Rs principle [37]: the possibility of being employed in multiple experiments, for different research fields, replaces the use of in vivo animal models for the experimental phases immediately following the in vitro experimentation (replace). A direct consequence of this is a reduction in the number of animals used during the experimental period (reduce). Finally, the use of a CAM assay allows researchers to minimize animal suffering, since the chick embryo does not exhibit nociception until day 11 of embryonic development (refine), as established by the National Institute of Health [38] as well as the Institutional Animal Care and Use Committee [39]. In addition, pain perception does not fully occur before the 15th day of embryonic development due to the immaturity of the portion of the central nervous system devoted to pain perception.

This review provides an overview of the uses of CAM assays in the two last decades and suggests the use of in ovo tests as an alternative to animal testing in preclinical studies; this could be a good solution to use in the field of regenerative medicine as a model for testing therapies for the resolution of critical bone fractures.

## 2. The CAM

**Figure 1:**
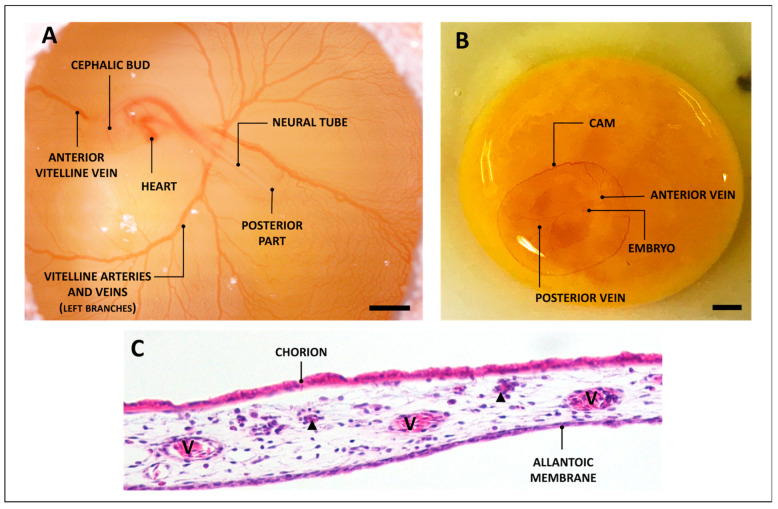
(**A**) Observation of a chicken embryo at ED3 (scale bar = 1 mm). (**B**) Chicken embryo at ED3 in an ex ovo experiment (scale bar = 0.5 cm). (**C**) Hematoxylin and eosin staining of a chicken CAM at ED13; image taken using a Nikon microscope at 10× magnification. V: a major CAM blood vessel; arrow-head: sub-chorion capillaries.

The chicken embryo’s chorioallantoic membrane (CAM) is a highly vascularized extraembryonic structure that functions by exchanging gas and nutrients for the embryo during the entire period of its development; further, it is also responsible for calcium mobilization from eggshell to promote embryonic bone mineralization [40,41,42]. It originates from the fusion between the mesodermal layer of the allantois and the adjacent mesodermal layer of the chorion, forming a double layer structure with a rich vascular network (Figure 1C), connected with the embryonic circulation by two allantoic arteries and one allantoic vein [43]. The mature CAM morphologically resembles a “C” and is permeated by a fluid called allantoic fluid, which physiologically provides nutrients and carries waste substances out of the embryo [44,45]. Thanks to its features, CAM is a convenient and versatile biological instrument.

The timing of the embryo’s development was described by Hamburger and Hamilton in 1951, and was subdivided in 46 chronological stages (HH stages) by using specific characteristics that occur at each step of the chick’s development regardless of the exact age of the embryo [46]. However, chicks’ developmental time can be difficult to assess because the egg is internally fertilized and goes through a brief period of development; for this, actual incubation days are usually considered for experimental dating, assuming that embryonic development begins for all eggs simultaneously at the time of incubation at a constant temperature and humidity.

The CAM begins its formation at about day 3 of embryonic development (ED3) and reaches completion around ED9, which is precisely why its use requires that tests be set up no earlier than ED 8–9 [47]. No CAM experiments reach the hatching period of the eggs, which, for chickens, is set at day 21 of embryonic life; however, experimentation can be carried out either in ovo or ex ovo, Latin for “inside the egg” and “outside the egg”, respectively. Initially for both methods, fertilized eggs from avian species (i.e., quail, turkey, and duck [48,49,50,50,51,52,53,54]), most commonly chicken, are kept in a humidified incubator at a constant humidity of 45% and a temperature of 37 °C for up to 15 days, by which time experimentation is allowed without the need to seek ethics committee approval. Indeed, as stated earlier, as sanctioned by Institutional Animal Care and Use Committee (IACUC), the chicken embryo is not considered a living organism until the 17th day of embryonic life [38,39].

Within this two-week period, certain operations are performed to make the egg accessible: opening, insertion of materials/molecules to test, final observation, and tissue collection [55]. In the case of the ex ovo technique, at ED3, the eggshell is cracked and its contents are placed in a sterile container. Several authors have developed various methodologies: eggs in cubes [56], the use of petri plates [57], the use of plastic glasses with water [58]; weighing trays [59], etc. In the case of in ovo experimentation, at ED3, the egg can be opened by making a small window to access the shell after aspirating a few ml of albumen from the opposite pole to the air chamber, in order to preserve the CAM during the shell access procedure. In any methodology chosen, in the opening phase, the operator visualizes the viability of the embryo by verifying the palpitation of the heart and observing the embryonic morphology characterized by the presence of a cephalic bud, posterior part, neural tube, and the classic “spider” structure of the vessels disposed around the embryo and composed of the main veins and arteries feeding the embryo (Figure 1A). The CAM is planar and can be easily observed as a circled area highly vascularized around the embryo (Figure 1B). 

The CAM is considered to lack immune competence until ED16–17. In fact, the lymphoid cells (i.e., mononuclear phagocytes, T and B cells) are present from ED11–12, but they are immature; therefore, the immune system is not active [60,61,62,63,64,65]. Since the CAM has a very dense capillary network, it is commonly used to study both new vessel formation (angiogenesis versus vasculogenesis) and its inhibition in response to different factors. 

The main advantages of CAM include: low cost, function as a natural bioreactor and well-vascularized system; high reproducibility and reliability; the natural immune deficiency of non-sentient embryos, and no need for ethical approval (until ED17). On the other hand, as with all models, CAM displays some disadvantages: short time to allow for cell migration, similarity between pre-existing and newly-generated vessels, susceptibility to environmental changes and contaminations by fungi and bacteria, different metabolism with respect to humans, need for skill and practice to handle the model before hatching, and rapid morphological modifications during embryonic development. Other limitations of the CAM method include the variations due to the fertilization rate in different seasons, and some difficulties particularly related to ex ovo procedures and peculiar applications. 

However, in recent years, the use of CAM has been greatly expanded, and there are now many applications.

## 3. The Application of CAM up to Now

The vascularized environment of the CAM of avian species offers the possibility to study a variety of molecules and materials; in particular, a CAM assay can be used to test pro/anti-angiogenic potential, to perform cancer studies, to test molecules and materials [66,67], to verify transplant reactions, and to test some drug effects. 

The clinical research in the field of regenerative medicine typically involves an initial phase of in vitro testing, including tests of cytotoxicity, biocompatibility, and others, but some analyses need to be confirmed using animal models. In this situation, CAM can be used as an alternative to animal experimentation, since it is comparable to a natural “in ovo” bioreactor. This makes future research more sustainable and makes it possible to lower the costs of the pre-clinical phase and speed up the preliminary tests needed prior to animal testing (which is currently mandatory for product entry into the clinical phase). 

The following paragraphs provide a brief overview of CAM’s uses for various experiments conducted in the last decade. Next, we will differentiate the past use of CAM with the perspective uses that can be made of it, such as the set-up, for example, of organotypic cultures [16].

### 3.1. Use of the Cam Assay for Cancer Studies 

Jankovic B.D. et al. were among the first to assert that CAM, as a highly vascularized membrane, together with extracellular matrix (ECM) proteins, mimics the physiological environment of cancer [30]. Thus, the CAM assay is considered particularly suitable for studying the distinctive aspects of cancer, such as angiogenesis, invasion, metastasis formation, and cancer cell spread [68,69,70,71,72]. There are various advantages and disadvantages that make the use of CAM versus the use of animal models an alternative way to study tumors.

One advantage that the CAM model has over the animal model in the study of tumor invasion is the time required for the development of visible microtumors, which, in animal models, become evident only several weeks after cell transplantation, whereas in the CAM model, tumor growth can be observed as early as a few days after cell grafting [73].

The short timing of embryonic development is another benefit of using CAM, because it is possible to speed up and simplify data collection in the pre-clinical phase. Indeed, the entire period of embryonic development is faster compared to that of any animal model, and this allows for rapid morphological feedback, for example, on the development of the vascular network in response to different types of grafts. At the same time, the difficulty of distinguishing newly formed blood vessels from preexisting ones is the main disadvantage of using CAM in cancer research. Finally, among the disadvantages of using the CAM assay, there is the difficulty of maintaining a sterile system and avoiding environmental contamination [74].

As for its strengths, which are more numerous than its disadvantages, the CAM model is widely used for tumor grafting, which can be implanted on the membrane in various forms: patient-derived xenografts; solid biopsies; circulating cancer cells in suspension; or, most commonly, tumor cell lines. Patient-derived xenografts retain many of the biological features of primary tumors, and, therefore, by grafting them onto CAM, it becomes possible to investigate the genetic, protein, morphological, and pharmacological patterns, as well as cancer-specific immune evasion mechanisms [73,75,76,77]. By transplanting biopsies of mammalian tumors, it is possible to maintain the main features of primary tumors and to perform studies regarding cell polymorphisms, mechanisms of growth and angiogenesis, interaction with the extracellular matrix, and metastasis formation [78,79,80]. Grafting circulating tumor cells is useful for analyzing the aggressiveness and proliferation ability of primary tumors, with the aim of performing pre-clinical drug screening and discovering biomarkers [77].

Table 1 shows some of the several cell lines which have been implanted in CAM in the last decade.

### 3.2. Cancer Hallmarks Studied in CAM: Angiogenesis

The CAM model has been widely used in the past to study the hallmarks of cancer, such as angiogenesis, proliferation, and tumor invasion, as well as to analyze the conditions underlying cancer therapies [100]. The process of developing a vascular network that supplies nutrients and oxygen to tumor cells has obviously been the subject of multiple studies, because growing tumors take advantage of the host’s physiological angiogenesis and promote its exuberant development to secure adequate oxygen and nutrient supply, to dispose of waste products, and to facilitate the dissemination of tumor cells to other districts [101].

Tumor onset and progression take place in successive phases, one of which is the avascular phase, during which an “angiogenic switch” can be triggered, resulting in vascular branching and endothelial cell proliferation and allowing the tumor to grow while ensuring a sustained energy supply [102]. In this way, even an initially benign neoplasm can evolve and mutate, and vascular proliferation can allow for its development beyond its benign dimensions. The deregulation of angiogenesis is also an hallmark of cancer [103], and inhibition of the altered tumor angiogenic process has been utilized as a therapeutic strategy for a long time now [104].

After cell grafting on CAM, tumors become visible within 2–3 days and are readily supplied with CAM-derived blood vessels that penetrate deeply into the tissue. Several qualitative and quantitative approaches have been employed to assess the angiogenic response to different types of treatments [69].

Demcisakova Z. et al. validated angiogenetic potential by immunohistochemistry against embryonic endothelial markers such as WGA (wheat germ agglutinin) and SMA (smooth muscle actin), chicken-specific myofibroblast (α-SMA). Monoclonal antibodies specifically recognize chicken monocytes, macrophages, and interdigitating macrophage cells (KUL01); moreover, with RT-PCR, it is possible to quantify the gene expression of angiogenesis markers such as VEGF (vascular endothelial growth factor), FGF-2 (fibroblast growth factor-2), ANG-1 (angiopoietin-1), and HIF-1α (hypoxia-inducible factor 1-alpha) [105].

In other studies, the formation new vessels has been quantified through immunohistochemistry to chicken-specific CD34 (predominantly regarded as a marker of hematopoietic stem cells and hematopoietic progenitor cells) or using a particular lectin (biotinylated lens culinaris agglutinin) that binds specifically to endothelial cells of chicken veins, arteries, and capillaries. That hybridization was used to assess the angiogenesis that is generated at the intra-tumoral level after grafting osteosarcoma cells onto CAM [71,106]. The tumor supply system has been the object of many studies in which anti-angiogenic drugs and biomaterials have been used to slow down the tumor growth process. Some of these anti-angiogenic drugs tested in ovo have been molecules that inhibit the VEGF and the platelet-derived growth factor receptors [107,108,109]. Another anti-angiogenic mechanism tested in CAM involves microRNAs (miRNAs) that play a key role in gene expression [110,111]. Among these, microRNA-21 (miR-21) is an oncogenic miRNA [110], the overexpression of which can downregulate key tumor inhibitory proteins, such as programmed cell death protein [112], TNFα (tumor necrosis factor-α), ERK (extracellular signal-regulated kinase), and VEGF [113]. miRNA-based therapy can be considered as a knockdown of miR-21 expression, induction of tumor cell apoptosis, and suppression of tumor-associated angiogenesis [114,115].

Finally, in a study conducted by Tome Y., another strategy was tested in human osteosarcoma cells transplanted onto CAM, involving the echistatin. This cyclic peptide functions as an anti-angiogenic molecule by bonding to the integrin α v β 3, thus inhibiting it [116].

### 3.3. Cancer Hallmarks Studied in CAM: Metastatic Potential

For several years now, the CAM model has been recognized as a viable alternative to animal models for the characterization of tumors and for their metastatic potential [117]. The CAM model also allows for the potential development of metastases in all organs of the chicken embryo [73]. Along with this feature, the intrinsic aggressiveness of various tumor forms was found to have more explanatory elements in diagnostic and therapeutic phases. In this context, by supplying the chicken embryo’s circulatory system with blood and nutrients, CAM provides an ideal system by recreating the physiological microenvironment for the cell–cell and cell–matrix interaction studies that occur during the metastatic cascade [100]. After the injection of tumor cells into the circulatory system of the chicken embryo, metastatic potential can be assessed by tracking the mRNA levels of metastasizing cancer cells in chick embryos. Each metastatic site is analyzed from a morphological and invasive potential point of view [118]. Traditional morphological detection techniques used in animal models can also be used in the CAM model. Indeed, to identify cell morphology and location, tumor samples can be subjected to hematoxylin and eosin staining as shown by Shioda and coworkers, who detected colon cancer cells by labeling sections of embryonic organs with the anti-human pan cytokeratin antibody [118,119].

Cell invasiveness, moreover, can be monitored by labeling tumor cells with fluorescent molecules that allow for the detection of scattered cell colonies in the various embryonic body districts, and simultaneously labeling chicken blood vessels with a particular lectin (biotinylated lens culinaris agglutinin) [120,121]. Ranjan R.A. and his team compared two breast cancer cell lines, MCF-7 and MDA-MB 231, to study growth rates by morphological evaluation, proliferation by immunohistochemistry for the Ki-67 protein, aggressiveness by evaluating the mitotic rate and tumor budding, and, finally, cell spreading using the Alu-PCR assay [68].

The latter involves specific in situ hybridization of the repeated sequences in a human genome named Alu, which are present only in humans with a frequency of 5% [122,123]. Tissue sections are subjected to RT-PCR for Alu sequences and CR1 (Chicken Repeat-1) to make a quantitative assessment of the human tumor cells intravasating and disseminating into the chick embryo through the CAM. It also, at the same time, distinguishes human cells from chicken vessels [69,71,73,97,117,119,124,125,126]. The search for human gene sequences by RT-PCR for a certain determined gene has been a technique used for several years now; in fact, many years ago, Kobayashi and coworkers identified metastatic prostate cancer cells disseminated in the liver and femur of a chicken embryo by amplification of the human beta globin gene [127].

### 3.4. Tumor Therapy Test in CAM

The CAM model is a versatile, yet also relatively simple and low-cost, model that also allows for the screening of pharmacological or physical therapies within a short time. Moreover, the use of the CAM model can be considered a precision tool for medicine to be used in the search for tailored cancer drugs [128]. Drugs that inhibit tumor growth have been tested in CAM in two main ways: by injecting them into the chicken’s circulatory system or by using them as a treatment of tumor cells, seeded appropriately on CAM, as reported in the protocol developed by Kunz and his team [71].

Therefore, the CAM assay is a reference model for several therapeutic approaches, including various chemotherapeutics [86,97], targeted [129,130,131] and checkpoint therapies [132], oncolytic viruses [133], radiotherapy [134], molecules that block the cell cycle and induce apoptosis [135,136], and anti-angiogenesis drugs [137,138].

## 4. Use of the CAM Assay to Validate Scaffolds for Regenerative Purposes

Regenerative medicine, in recent years, is progressing toward new translational approaches based on the formulation and fabrication of Advanced Therapy Medical Products (ATMPs). It is, therefore, tissue engineering (TE), a branch of research that aims to produce constructs that are the results of a combination of cells, biomaterials, and biologically active molecules, in the form of scaffolds with the aim of repairing tissues by inducing their regeneration [139,140,141,142,143,144,145,146]. TE can be conducted ex vivo or in situ [147,148]: the first approach requires the seeding of donor stem cells onto a scaffold that is inserted into the affected tissue for the purpose of stimulating cell growth and differentiation [149,150,151,152]; the in situ method, on the other hand, avoids the step of seeding cells onto the scaffold and involves the fabrication of scaffolds that can adapt to tissue damage in terms of their size and shape. The latter contain biocompatible materials that can be implanted at the site of damaged tissue, where they attract the surrounding host cells necessary for healing to the repair site [145,147,148,153,154]. Specifically, among the components of TE constructs emerge biomaterials which hold many key characteristics for in vivo implantation into host tissues. These include biocompatibility to avoid the induction of an immune response, sterilizability to be safely incorporated into host tissues, biodegradability to be degraded by tissue cells into easily metabolized molecules from the tissue after performing their function, and bioactivity to stimulate tissue repair. Regardless of biochemical composition and biophysical properties, their most important feature is the interaction with the biological system in which they are embedded [155,156].

Biomaterials can be classified according to their origin. There are those of natural origin, such as chitosan, alginate, and cellulose [157,158], and those of synthetic origin, such as PLGA poly (lactic-co-glycolic acid), PCL (lactic-co-glycolic acid), PLA (polylactic acid), fibronectine, and polyurethane [158,159].

In this context, once again, the CAM model represents a natural bioreactor which can be used to test the main characteristics of biomaterials and the effects they have on the CAM, which represents a viable system. In recent years, the CAM assay become a popular approach in tissue engineering studies, in particular in the study of different tissue pathologies, such as those related to bone defects [64,160]. The chorioallantoic membrane allows for the observation of the effect that biomaterials have on the angiogenesis and tests their biocompatibility. Considering the central role that angiogenesis plays in tissue regeneration, the evaluation of the angiogenic potential of biomaterials has become a priority in TE, especially for bone TE [161,162,163,164,165].

The angiogenic potential and biocompatibility of several biomaterials have already been tested in CAM. Many of the biomaterials which have been tested in CAM are of synthetic origin; among them are hydrogels, which mimic extracellular matrix materials (ECM) due to their highly hydrated, permeable, and porous structures. They enable guided tissue regeneration by facilitating cellular activities, nutrient diffusion, and waste transfer [166]. The swelling and degradation ability of polymer matrix hydrogels makes them suitable for encapsulating and delivering numerous therapeutic agents, such as cells, growth factors, drugs, and genes, into tissue defects [167,168]. In addition, hydrogels are very often enriched with other molecules, such as, for example, acrylamine [169], heparin [170], and hyaluronic acid [171]. The latter is designed to treat periodontitis, a chronic biofilm-associated inflammatory disease of the tooth-supporting tissues that causes tooth loss. The scaffold developed by the team, based on controlled oxygen-releasing hyaluronic acid, is useful in avoiding a hypoxic environment that would compromise tissue regeneration [172,173]. Other biomaterials tested in CAM include bioplastics, which are eco-friendly materials used in bone tissue regeneration for their biocompatibility and biodegradability. Specifically, poly (3-hydroxybutyrate-co-3-hydroxyhexanoate) (PHBH) reinforced witch cellulose nanocrystals (CNCs) has been tested in CAM in the form of a porous scaffold. The CAM assay enabled the identification of the scaffold pore size, which is more optimal for endothelial cell colonization and blood vessel formation [67]. Bioactive glasses, in the TE field, have also received a significant amount of interest. These, enriched with biologically active ions of various kinds, such as boron, were the focus of research conducted by Decker and coworkers. They observed the influence of B-doping of bioactive glasses on the viability, osteogenic differentiation, and expression of osteogenic and angiogenic marker genes of bone marrow-derived mesenchymal stromal cells (BMSCs), in the presence of the B-BGs’ ionic dissolution products (IDPs); subsequently, they evaluated the influence of IDPs on chorioallanotic membrane angiogenesis [174]. In the same way as bioglass, synthetic hydroxyapatite (HA), which is a particular type of calcium phosphate, has been widely examined as a regeneration material because of its affinity to the main natural component of bone and its osteoconductivity and bioactivity [175,176,177,178,179]. In this regard, HA formulated with other biomaterials, such as biopolymers, demonstrates remarkable vasculogenesis, as is evident from CAM testing conducted in recent studies focused on finding viable regenerative strategies for the orbital floor [159]. Other biopolymers also fit into this context: Demsisakova et al. developed a scaffold consisting of the biopolymer polyhydroxybutyrate (PHB) combined with chitosan (CHIT). Also, using the CAM assay, they demonstrated that (PHB)/(CHIT) has strong endogenous angiogenic potential and could be a promising biomaterial for the treatment of hard tissue defects [105]. The most significant advantage of using CAM in studying the properties of biomaterials is that the CAM allows for the development and branching of the vascular network on the implanted scaffolds, mimicking what should occur in tissue in vivo. In this regard, the porosity and pore size of the scaffold play key roles in vascular infiltration and osteogenic differentiation [180,181,182]; therefore, the challenge for researchers seems to be to formulate ever-new constructs that have better porosity and efficiency in TE.

## 5. Use of CAM to Set-Up Organotypic Culture

All the advantages of CAM also make it an attractive model for tissue engineering approaches. The membrane, during the developmental stages of the chick embryo, provides a naturally immunocompromised host and a rapidly growing vascular bed that lacks a nervous system and, therefore, provides a less sentient alternative for animal research. This allows for in vivo implantation of organs and represents a model for xenografts developing organotypic cultures [73,183]. This provides a solution to the most important limitation of the ex vivo organotypic culture: the lack of blood, immunity cells, and bone cells [184,185].

Given that, especially in the field of bone tissue engineering, it is possible to use both the embryo and the membrane itself, Blake et al., on the 18th day of embryo development, harvested the femur of the embryo, which they then implanted onto the CAM after causing a fracture [184].

As shown by Aldamash A. et al. [186] and Marshall et al. [187], the use of the chicken embryo femur also had another application; the works of these two researcher groups aimed to examine the differentiation potential of specific cells, such as human bone marrow stromal cells (HBMSCs) and human neonatal foreskin stromal cells (hNSSCs), alone or in combination with human umbilical vein endothelial cells (HUVECs), under experimental conditions for tissue regeneration. The authors took the chicken embryo femur, caused a fracture, and cultured it with different cell types to test their differentiation potential as well as, in the case of hNSSCs and HUVECs, their angiogenic potential.

Although the CAM model per se allows for short experimental times (from day x to day y), it is also possible to overcome this time limit by performing transplantation from one chorion allantoic membrane to another ex vivo of organotypic cultures or biomaterials to be tested on CAM; of course, in case of the need for increased experimental time, one must be careful not to damage the grafted samples/scaffolds in transport from one CAM to the other. In a recent study, Feder et al. set up a protocol whereby it is possible to graft onto the CAM various sections of osteosarcoma tissue taken from rats and mice; then, they transplanted them onto another membrane five to seven consecutive times, enabling further experimental analyses [188].

Another insight about CAM’s applications in this field is provided by Kanczler and his team, who devised a critically-sized chick femur defect model, which has been used to evaluate different types of molecules engaged in bone tissue engineering. Specifically, CAM is used to test the effects of different factors and proteins involved in healing bone defects, such as bone morphogenetic protein-2 (BMP-2), vitamin D3, parathyroid hormone (PTH), and parathyroid-hormone-related protein (PTHRP), to assess the potential of osseointegration of scaffolds and to evaluate their performance before using them in in vivo studies [189,190,191].

Other studies report how CAM can also be used for transplanting sections of organs or organoids from murine embryos, such as, for example, the kidney. Embryonic kidneys were taken at ED11.5, transplanted onto CAM at ED8, and then cultivated for 7 days; subsequently, the murine embryonic kidney, grown on CAM, underwent immunohistochemistry for endothelial growth markers, highlighting the anastomosis between the blood vessels of CAM and those of the murine kidney [192].

## 6. Discussion and Conclusions

As described above, there is ample evidence that the use of CAM in numerous research areas is effective for research and clinical studies. A positive fact is that the CAM model, inspired by the 3R concept, is a viable alternative to classical animal experimentation, which is no longer sustainable without ethical limitations/controls and which, in the future, will have to be replaced with (or must be accompanied by) alternatives that are more advantageous in economic, ethical, and experimental terms. It has already been pointed out that research in the field of oncology and into biomaterials for regenerative medicine are highly developed areas, in which more resources have been invested in the development of techniques and strategies for the use of CAM corresponding to the demands of research. In addition, other applications of CAM are cautiously being developed in order to use this powerful vascularized natural bioreactor for the accomplishment of organ cultures, which could replace the early stages of experimentation and are currently performed exclusively on animal models, thus decreasing the negative impact of research on animal welfare.

This review has highlighted the most significant scientific studies from the last decade to underline the current importance of this alternative model to animal experimentation. Finally, we would like to point out that the interest in the use of CAM has recently materialized, with the 1st International Conference on CAM held in February 2022, which brought together highly prestigious scholars from all over the world.

## 7. Future Directions

In the future, it is to be hoped that even more standardized techniques will be developed for setting up CAM testing services for large research macro-areas in order to meet all scientific demands. In addition, more publicity should be given to this alternative model to in vivo testing due to all of the advantages and properties described above. The possibility of setting up organ cultures on CAM is attractive, useful, and innovative. At present, the use of this tool is not widespread, and the development of methodologies for the use of CAM for organ cultures is slow. In the coming years, it is hoped that more and more research groups will devote themselves to the implementation of this organ culture model in order to test various molecules and clinical strategies from a translational perspective.

## Figures and Tables

**Table 1:** Tumor types tested in CAM in the last six years.

Organs	Research Papers
Prostate	[81,82]
Colon	[71,83]
Pancreas	[84,85,86,87]
Breast	[88,89]
Lung	[90]
Glioblastoma	[91,92]
Osteosarcoma	[71,93,94,95]
Retinoblastoma	[86,96]
Neuroblastoma	[97]
Malignant Pleural Mesothelioma	[98]
Ovary	[99]

## Data Availability

No new data were created or analyzed in this study. Data sharing is not applicable to this article.
